# The Quality of the Evidence According to GRADE Is Predominantly Low or Very Low in Oral Health Systematic Reviews

**DOI:** 10.1371/journal.pone.0131644

**Published:** 2015-07-10

**Authors:** Nikolaos Pandis, Padhraig S. Fleming, Helen Worthington, Georgia Salanti

**Affiliations:** 1 Department of Hygiene and Epidemiology, Medical School, University of Ioannina, Ioannina, Greece; 2 Barts and The London School of Medicine and Dentistry, Queen Mary University of London, Turner St., London E1 2 AD, United Kingdom; 3 Cochrane Oral Health Group, School of Dentistry, The University of Manchester, Coupland 3 Building, Oxford Road, Manchester, M13 9PL, United Kingdom; 4 Department of Orthodontics and Dentofacial Orthopedics, Dental SChool/Medical Faculty, University of Bern, Bern, Switzerland; Copenhagen University Hospital, DENMARK

## Abstract

**Objectives:**

The main objective was to assess the credibility of the evidence using Grades of Recommendation, Assessment, Development, and Evaluation (GRADE) in oral health systematic reviews on the Cochrane Database of Systematic Reviews (CDSR) and elsewhere.

**Study Design and Setting:**

Systematic Reviews or meta-analyses (January 2008-December 2013) from 14 high impact general dental and specialty dental journals and the Cochrane Database of Systematic Reviews were screened for meta-analyses. Data was collected at the systematic review, meta-analysis and trial level. Two reviewers applied and agreed on the GRADE rating for the selected meta-analyses.

**Results:**

From the 510 systematic reviews initially identified 91 reviews (41 Cochrane and 50 non-Cochrane) were eligible for inclusion. The quality of evidence was high in 2% and moderate in 18% of the included meta-analyses with no difference between Cochrane and non-Cochrane reviews, journal impact factor or year of publication. The most common domains prompting downgrading of the evidence were study limitations (risk of bias) and imprecision (risk of play of chance).

**Conclusion:**

The quality of the evidence in oral health assessed using GRADE is predominantly low or very low suggesting a pressing need for more randomised clinical trials and other studies of higher quality in order to inform clinical decisions thereby reducing the risk of instituting potentially ineffective and/or harmful therapies.

## Introduction

Systematic reviews aim to assimilate high-quality evidence on the area of interest in a systematic, transparent, and unbiased manner leading to qualitative or quantitative synthesis. Quantitative synthesis can produce a more precise estimate on the efficacy and safety of a therapy, and can reconcile misunderstandings and controversies, and form the basis for future trials [1]. Healthcare practitioners should seek high quality evidence when searching for answers to clinical questions in relation to the effectiveness or otherwise of a proposed intervention. Several meta-epidemiological studies have been published on the quality of systematic reviews in oral health, with evidence to suggest both reporting and methodological deficiencies common to systematic reviews within a range of specialty areas [2–5]; these findings have also mirrored analogous research studies in other biomedical areas [6,7]. However, the overall quality of the existing evidence in oral health has not yet been assessed. Moreover, there is increasing concern of a gulf between research evidence and its clinical applicability. Patients are not typically conversant in the scientific publications but are exercised by the implications of research findings on their lives and wellbeing. It has become evident that a system capable of simultaneously assessing the quality of the evidence, balancing benefits and harms, while accounting for patient preferences and aiding clear treatment recommendations is imperative [8]. This approach would resonate both within medicine and in dentistry where an increasing number and quality of systematic reviews are published.

Several groups have proposed complex methods for evaluating and translating evidence into clinical practice; many of these have been somewhat confusing and impractical [9]. The GRADE (Grades of Recommendation, Assessment, Development, and Evaluation) initiative, however, has become an accepted approach for assessing the evidence and consequently making recommendations [8]. The GRADE approach is predicated on a precise clinical question, with consideration of all important outcomes within a systematic review prioritizing them based on their relative importance [10]. Subsequently, the existing quality of the evidence for an intervention and a particular outcome from a systematic review is assessed based on a specific protocol and graded as high, moderate, low or very low (Table 1) [11].

**Table 1:** Categories of quality of evidence according to GRADE. A single downgrade (for randomized clinical trials) or upgrade (for non-randomized studies) corresponds to one level change in the ranking.

Rank	
**High ++++**	Further research is very unlikely to change our confidence in the estimate of effect
**Moderate +++**	Further research is likely to have an important impact on our confidence in the estimate of effect and may change the estimate
**Low ++**	Further research is very likely to have an important impact on our confidence in the estimate of effect and is likely to change the estimate
**Very Low +**	Any estimate of effect is very uncertain

While, precursors relied heavily on the overall study design (randomized compared to non-randomized studies) to evaluate a body of evidence, study design remains important within the GRADE assessment, although not the sole arbiter of the quality of evidence. Randomized clinical trials provide a higher quality of evidence compared to observational studies; however, good quality observational studies are more valuable than uncontrolled case series designs [12]. Randomized clinical trials (RCTs) are initially rated as high quality but may be downgraded after accounting for study limitations, indirectness, inconsistency, imprecision, and publication bias. Observational studies start at low quality but may be upgraded in the presence of a large magnitude of effect after potential confounding and dose-response effects have been considered. In the GRADE system, although expert opinion does not command a quality of evidence score, it is considered critical in interpreting, combining and placing the available evidence in the correct context [12].

The GRADE approach has been endorsed by a large number of societies and institutions including The Cochrane Collaboration [13] and has been used in various fields of medicine such as allergies, heart disease, oncology, endocrinology and respiratory and critical care medicine [14–17]. GRADE has also been used in two small empirical studies to appraise the quality of evidence to support medical interventions in two medical areas [18,19]. While GRADE is applicable within the oral health field [20,21], there are no previous reports of an overall assessment of the quality of evidence in this area. Therefore, the aim of this study is to assess the quality of the evidence in contemporary oral health reviews using GRADE, and to assess possible differences in the quality of the evidence between Cochrane systematic reviews and non-Cochrane reviews.

### Methods

The following pre-specified inclusion criteria were applied for the systematic reviews and meta-analyses:

Eligible systematic reviews or meta-analyses for inclusion in this study included at least one meta-analysis of at least two original studies; only one meta-analysis per systematic review was considered.

The selected meta-analysis was that reporting on the primary outcome or the first or most important reported outcome if the primary outcome was not specifically outlined.

If meta-analysis of more than one primary outcome was available, the meta-analysis including the largest number of trials was selected.

The following pre-specified exclusion criteria were applied:

- Systematic reviews with forest plots without a pooled estimate.
- Duplicate publications, laboratory studies and reviews of animal studies.
-Quantitative synthesis within single arms either due to absence of a control or due to analysis of before-after measurements.
- Reviews including meta-analysis, which included the same studies multiple times without explanation on whether subgroups were mutually exclusive.
- Systematic reviews or reviews using network meta-analysis.
- Diagnostic test accuracy reviews or reviews on prognostic factors.

Systematic reviews or meta-analyses published from January 2008 (year of GRADE adoption by the Cochrane Collaboration) until the end of 2013 were retrieved from the Oral Health Group (OHG) of the Cochrane Database of Systematic Reviews (CDSR) and by hand searching of 14 general and specialty dental journals (January 2008-December 2013) with the highest impact factor (IF) in 2012 (Table 2).

**Table 2:** Frequencies and percentages of included systematic reviews per journal type and journal impact factors.

		Impact factor	N (%)
**Journal**	American Journal of Orthodontics and Dentofacial Orthopedics (AJODO)	1.46	5 (5.5%)
	Caries Research (CR)	2.51	1 (1.1%)
	COCHRANE Database of Systematic Reviews: Oral Health Group (CDSR-OHG)	5.79	41 (45.1%)
	Clinical Implant Dentistry and Related Research (CIDRR)	3.82	4 (4.4%)
	Clinical Oral Investigations (COI)	2.20	3 (3.3%)
	Clinical Oral Implant Research (COIR)	3.43	5 (5.5%)
	International Journal of Prosthetic Dentistry (IJPD)	1.63	1 (1.1%)
	Journal of Clinical periodontology (JCP)	3.69	7 (7.7%)
	Journal of Dentistry (JD)	3.20	4 (4.4%)
	Journal of Dental Research (JDR)	3.83	4 (4.4%)
	Journal of Endodontics (JOE)	2.93	3 (3.3%)
	Journal of Oral and maxillofacial Surgery (JOMS)	1.52	6 (6.6%)
	Journal of Periodontology (JP)	2.40	7 (7.7%)
Total			91 (100%)

The titles and abstracts were initially read by one investigator (NP) and all full-text articles were retrieved and screened for inclusion. A second screening of the full reports was undertaken by one investigator (NP) resulting in exclusion of the reviews from further analyses based on the pre-specified exclusion criteria. Information was collected from all selected review articles at the review, meta-analysis and trial level (S1 Table).

The conclusions of selected meta-analyses were assessed using the GRADE approach by the first author, who was familiar with GRADE. A second author (PSF) verified the ratings; any disagreements were reconciled after discussion. The selected meta-analyses were assessed in relation to the quality of the evidence scored in the 5 domains specified within GRADE: Limitations in study design and/or execution (risk of bias) [12], inconsistency of results [22], indirectness of evidence [23], imprecision of results [24], and publication bias [25]. The overall GRADE rating results based on 4 levels (high, moderate, low and very low) are shown in Table 1. A more detailed description of the GRADE process is shown in the appendix (S1 File and S2 Table). The GRADE judgment for Cochrane reviews was re-assessed independently of the findings of the original review authors.

## Data Analysis

The objectives of the statistical analyses were to tabulate frequency distributions of specific characteristics in the dental systematic review sample at the review, and meta-analysis levels together with the frequency distributions of the quality of the evidence in relation to GRADE for Cochrane systematic reviews and non-Cochrane reviews (or meta-analyses). Associations between systematic review/meta-analysis characteristics and GRADE assessment were also considered. The four-level GRADE rating (high, moderate, low, very low) was converted into a binary variable (high/moderate and low/very low) in order to fit a logistic regression model to assess potential associations between GRADE rating, impact factor and publication year. GRADE was the dependent variable and impact factor and publication year were the examined predictors. All the analyses were performed with Stata statistical software version 13.1 (Stata Corporation, College Station, Texas, USA).

## Results

**Fig 1:**
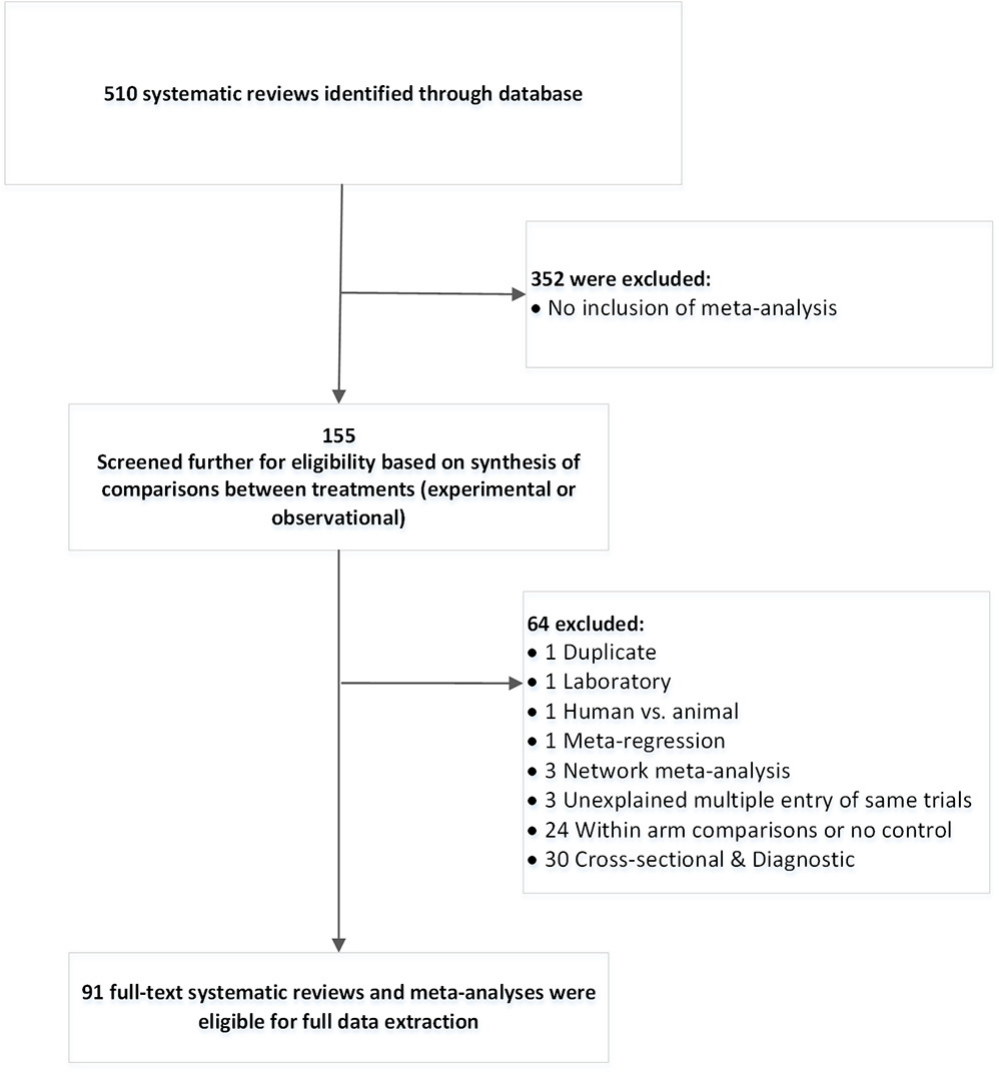
Systematic review selection flow diagram.

From the 510 reviews initially considered for inclusion in the study, 91 were included in the final assessment (Fig 1). The frequencies of reviews per journal are outlined in Table 2. Fifty reviews were included from the selected dental journals and 41 systematic reviews from The Cochrane Database for Systematic Reviews; a variety of conditions, interventions and outcomes were considered. The number of published systematic reviews with meta-analyses increased over time (S1 Fig). Significant differences were found between Cochrane and non-Cochrane reviews in respect of the region of authorship, involvement of a methodologist, number of collaborating centers, inclusion of GRADE assessment and assessment of harms in the outcome category. The Cochrane compared to non-Cochrane reviews are more likely to originate in Europe (OR: 14.62, 95% CI: 1.76, 121.19, p = 0.01) or Asia (OR: 1.43, 95% CI: 0.11, 18.00, p = 0.78) compared to Americas, to involve authors across multiple centers (OR: 5.26, 95% CI: 1.97, 14.09, p = 0.001), to include a methodologist (OR = 73.76, 95% CI:9.79, 555.93, p<0.001) and a GRADE assessment (OR = 12.71, 95% CI:4.17, 38.69, p<0.001), and to consider at least one harm in the outcomes (OR = 2.66, 95% CI: 1.13, 6.27, p = 0.03) (S3 Table).

The Cochrane systematic reviews included only randomized clinical trials but the non-Cochrane reviews included both randomized and non-randomized studies and occasionally a combination of both designs. Authors of Cochrane reviews were more likely to account for clustering effects (OR: 15.62, 95% CI: 2.81, 86.76, p = 0.002) often encountered in dentistry either in the analysis or discussion and to correctly analyze paired data (OR: 11.25, 95% CI: 11.05, 541.20, p = 0.02) compared to non-Cochrane reviews (S4 Table).

A variety of approaches were used for the assessment of the methodological quality with the Cochrane risk of bias tool used in 62 out of the 91 reviews. In some reviews, a combination of approaches was used and in some instances incorrectly reporting guidelines were utilized for this purpose.

The GRADE assessment indicated that only 2% of the evidence belongs to the high and 18% to the moderate category with the remaining reviews at the low/very low level (Table 3). The distribution of the overall GRADE ratings and ratings for each GRADE domain were similar for Cochrane and non-Cochrane reviews (Table 3).

**Table 3:** Frequencies and percentages of overall and per domain GRADE rating for Cochrane and non-Cochrane meta-analyses.

	Non- Cochrane Review	Cochrane Systematic Review	Total	p-value ^#^ ^,^ ^##^
	N (%)	N (%)	N (%)	
**GRADE rating**				
High	2 (4%)	0 (0%)	2 (2%)	ns
Moderate	9 (18%)	11 (27%)	20 (22%)	
Low	24(48%)	19 (47%)	43 (47%)	
Very Low	15(30%)	11 (27%)	26 (29%)	
**Domains**				
**Study limitations/Risk of Bias**				
No	2 (4%)	2 (5%)	4 (4%)	ns
Serious	25(50%)	30 (73%)	55 (60%)	
Very Serious	23(46%)	9 (22%)	32 (35%)	
**Inconsistency**				
No	34(72%)	31(76%)	65 (71%)	ns
Serious	15(26%)	10(24%)	25 (27%)	
Very Serious	1(2%)	0(0%)	1 (1%)	
**Indirectness**				
No	50(100%)	38 (93%)	88 (97%)	ns
Serious	0 (0%)	3 (7%)	3 (3%)	
Very serious	0 (0%)	0 (0%)	0 (0%)	
**Imprecision**				
No	19(38%)	19 (46%)	38 (42%)	ns
Serious	23(46%)	18 (44%)	41 (45%)	
Very Serious	8 (16%)	4 (10%)	12 (13%)	
**Publication bias**				
Suspected	8 (16%)	0 (0%)	8 (9%)	**
Unsuspected	42(84%)	41 (100%)	83 (91%)	
**GRADE rating as reported in the review**				
High	2 (4%)	0 (0%)	2 (2%)	
Moderate	1 (2%)	9 (22%)	10 (11%)	
Low	0 (0%)	10 (24%)	10 (11%)	
Very Low	2 (4%)	3 (8%)	5 (6%)	
Not reported	45(10%)	19 (46%)	64 (70%)	
**Total**	50 (100%)	41 (100%)	91(100%)	

*ns = non-significant*

** Significant at 0.*01*

*#Pearson X* ^*2*^ *test or Fisher’s exact test*

*## P-value based on High/Moderate vs Low/Very Low for GRADE and on No vs Serious/Very serious for the GRADE domain comparisons*

The most commonly downgraded domains were those for study limitations (risk of bias) and imprecision. The frequency of downgrading by 2 levels (very serious) was higher in the non-Cochrane reviews (46%) compared to the Cochrane reviews (22%); the former include also non-randomized studies. The inconsistency domain received similar ratings for non-Cochrane and Cochrane reviews in agreement with the reported statistical heterogeneity represented by *I* ^2^ (S2 Fig).

A GRADE assessment was included in 5/50 (10%) of the non-Cochrane and in 22/41 (54%) of the Cochrane reviews, respectively. Only three discrepancies were observed among the 27 included in the systematic reviews between the evidence rating found by the initial review authors and our re-analysis. One review published [26] in the Journal of Clinical Periodontology was based on a previous Cochrane systematic review which did not include a GRADE assessment [27]. Logistic regression analysis indicated that neither the journal impact factor (OR = 0.92, 95% CI: 0.68, 1.25, p = 0.61) nor the year of publication (OR: 1.17, 95% CI: 0.82, 1.67, p = 0.40) are important predictors for the quality of the evidence (Reference group: low/very low evidence).

## Discussion

This cross-sectional meta-epidemiologic study is the first attempt to evaluate the evidence across the oral health field and has exposed that only 2% of the existing evidence derived from reviews has a high level of evidence and only 18% was of a moderate level of evidence in relation to primary outcomes with no difference between Cochrane and non-Cochrane reviews. This finding indicates that 80% of the evidence considered, much of which will form the basis of clinical recommendations, is at the low or very low level. This finding is alarming indicating the need to undertake high quality randomized clinical trials in order to reduce the uncertainty around therapies in the oral health field. Empirical studies in oral health and medicine in general using GRADE to assess the quality of evidence are sparse [18,19]. Our study included 91 reviews and covered the entire field of oral health systematic reviews and meta-analyses; it is likely that the quality of the evidence may vary across, between and within healthcare specialties. Although, GRADE has been used to assess the quality of evidence within specific systematic reviews in several fields, we were able to identify only two small empirical studies across the range of biomedical fields where GRADE was applied in meta-epidemiological studies on a body of systematic reviews to assess the quality of the evidence within a particular specialty. The scope of those studies was limited to hyperbaric oxygen therapy indications [19] and nonsurgical treatment of stress urinary incontinence [18]. In the analysis of hyperbaric oxygen treatment[19], the quality of the evidence ranged from very low to high depending on the indication with key modifiers identified as risk of bias, imprecision and large effect for observational studies. Similarities with our study include reasons for downgrading the quality of the evidence based on risk of bias and imprecision. The review on nonsurgical treatment of stress urinary incontinence involved assessment of 13 reviews finding that the quality of the evidence ranged from low to high depending on the intervention [18].

Cochrane reviews are considered to be particularly rigorous and are conducted according to strict criteria and usually include only randomized clinical trials for assessment of benefits and harms and only use quasi-randomized studies and observational studies for the assessment of harms [2]. In the present study, the non-Cochrane reviews had a relatively small proportion of non-randomized studies; however, the quality of evidence did not differ among the groups suggesting the quality of studies populating both Cochrane and non-Cochrane reviews to be lacking. The two most frequently downgraded domains were risk of bias (study limitations) and imprecision. For the risk of bias (study limitations) domain lack of allocation concealment, lack of blinding, losses to follow-up and selective reporting commonly led to a downgrade [28]. In relation to the imprecision domain, small sample size and wide confidence intervals which include both benefit and harm are likely to prompt a downgrade [24]. Specifically, for binary outcomes, if the 95% confidence intervals ranged from unimportant to important benefits and from unimportant to important harms, the quality of evidence was downgraded when appreciable benefit or harm are of the order of 25% relative risk reduction or increase. For continuous outcomes, if the 95% confidence intervals included no effect and upper and lower confidence interval bounds crossed the minimal important clinical difference (MID) for harm or benefit downgrade was considered appropriate. If the MID was not known scores were downgraded if the upper or lower confidence limit crossed an effect size of 0.5 in either direction [24]. Downgrade for these reasons, in particular, were common as the number and size of the included studies was usually small. This finding was consistent with a recent study evaluating the quality of evidence in hyperbaric oxygen therapy [19]. The latter review, however, was also based on a relatively limited subset of 17 reviews, with the majority of primary studies (75%) being non-randomized studies.

In relation to the characteristics of the reviews, Cochrane reviews were more likely to be published by European authors, to involve authors across multiple centers, to involve a methodologist, and to consider at least a single harm in the outcomes than was the case for non-Cochrane reviews. However, the strict methodological and reporting criteria and the involvement of a methodologist did not result in higher quality of evidence. This is logical as, while Cochrane reviews are likely to be conducted and reported to a higher standard, published systematic reviews are based on studies from the same pool, regardless of the effect of methodological quality of the review itself, review authorship and associated expertise. This finding does not suggest that Cochrane systematic reviews are redundant with a level of commensurate with non-Cochrane reviews; Cochrane reviews are rigorous and replicable and this should be the standard moving forward.

The systematic reviews included a median of 5 meta-analyses, each comprising of a median of 5 studies. These figures are in keeping with a large survey of the Cochrane Database of Systematic Reviews involving analysis of 2,321 systematic reviews which highlighted a median of 6 meta-analyses per review and a median of 3 studies per meta-analysis [29]. A similar previous review [30] of dental systematic reviews reported a median of 9 studies were included in the largest meta-analysis within dental systematic reviews involving 9 dental specialties, with the largest meta-analysis having no more than 4 studies in 19% of reviews. Similarly, the largest meta-analysis involved a median number of just two randomized clinical trials, although that review referred back to systematic reviews from as long ago as 1991 when randomized clinical trials were considerably less prevalent in oral health than is now the case.

In oral health, due to the fact that multiple matched or unmatched sites can receive the intervention of interest, clustering effects are common. If this is handled improperly during the analysis, significant results may arise which are not genuine [31–33]. Additionally, a common design which uses matching called split-mouth studies requires consideration of the within patient correlations during the synthesis. In split-mouth designs, clustering effects can also exist when both interventions are applied within the same patient and in the presence of multiple sites per treatment arm as is the case with teeth [33, 34]. Studies with clustering effects, paired data or a mixture of these were handled more appropriately in the Cochrane systematic reviews than in the non-Cochrane meta-analyses.

The Cochrane Collaboration adopted GRADE in 2008; this may explain the inclusion of a GRADE assessment more frequently in the Cochrane Database of Systematic Reviews with 22/41 (82%) Cochrane systematic reviews including a GRADE assessment versus 5/50 (10%) among non-Cochrane reviews. Therefore, while Cochrane systematic reviews follow strict methodology, it is evident that deviations from the pre-specified ideal are possible. We conducted our own assessment of GRADE highlighting a discrepancy in only 11% of occasions with the rating of the systematic review authors confirming the reliability of the GRADE approach when assessing the evidence [35]. Other investigators found larger inconsistencies during the application of GRADE [36], although the level of familiarity of these investigators with GRADE is unclear. In the present study one of the investigators who assessed the quality of the evidence had attended a GRADE course and both GRADE assessors have implemented GRADE previously in systematic reviews.

Older systematic reviews, time from search to publication and delays in systematic review updates may lead to important differences in included studies compared to more recent reviews [37]. Publication year, however, was not found to have a bearing on the quality of the evidence in the present study. Similarly, journal impact factor showed no association with the quality of the evidence. There is some evidence that systematic reviews published in higher impact medical journals are of higher methodological quality [38]; however, this does not necessarily translate into higher quality of evidence. Systematic review methodological quality should not be confused with the quality of the evidence, although more detailed and broader searches in particular may lead to the identification of more eligible studies and potentially lead to higher quality of evidence.

The present study did not include a cross-section of all oral health reviews but focused on the Cochrane Database of Systematic Reviews and dental journals with the highest impact factor, which may have potentially influenced the results, although the pool of potential candidate studies for inclusion is similar. Nevertheless, the observed findings are likely to represent a best-case scenario. The inclusion of the highest impact factor dental journals possibly explains the lack of association between impact factor and evidence quality. The several steps of the GRADE assessment may introduce an element of inconsistency in the ratings; it is, therefore, important that the complete picture is considered along with the judgment made. Rating of study limitations for non-randomized studies can be problematic especially when the information provided in the review is limited. A particularly challenging area to rate is publication bias as it is difficult to detect exclusion of eligible studies. Both the description of the literature search, reference to grey literature, trial registries and reference lists of included studies during the assessment of publication bias were considered to reach this decision. Statistical assessment of publication bias was typically impossible due to the small number of included studies in the meta-analyses [39]. It is of interest to note that the Agency for Healthcare Research and Quality Evidence based Practice Center Program considers publication bias to be an optional domain [40]. Finally, evaluation of imprecision can be often challenging. The GRADE approach suggests the use of confidence intervals since dichotomous decisions based on statistical significance are problematic [24]. Alternative approaches that can be used to decide upon the conclusiveness of the evidence have been proposed more recently [41].

In the present study, one author performed the initial screening of the eligible systematic reviews and one author made the GRADE rating with scores verified by a second author. In a previous study [36] implementation agreement ranged from low to high depending on the domain and concluded that both training in GRADE and clinical expertise are instrumental in improving consistency in its use. GRADE permits judgment in a methodical and transparent way [42] with inconsistencies relating to the type and number of outcomes considered during the assessment [36]. The approach favors an overall rating which may be considered as a continuum hinging on expertise and judgment resulting in a judgment where the overall may not be the sum of all parts [42]. Nevertheless, as an incidental finding, significant agreement was observed between the ratings made in the constituent reviews and in the present cross-sectional study. For the purposes of assessing the association between GRADE rating and publication year and impact factor, and because data was thin the 4-level scale was converted to two levels. We understand that GRADE uses 4 levels for a purpose and distilling the conclusions into a 2-level scale has limitations. In this instance, however, the 2-level presentation helped in communicating the results especially when applying GRADE for recommendations.

This study dealt with only one outcome for which meta-analyses were available. A more comprehensive approach could have involved consideration of several or all outcomes and could have included evidence from qualitative synthesis. However, this would have been very difficult to implement given the large number of systematic reviews. Furthermore, we feel that inclusion of only the primary or first outcome over a wide range of oral health systematic reviews is likely to be a good proxy of the quality of the evidence in the field of oral health research.

In conclusion, only a small proportion (20%) of the studies assessing interventions in oral health was of moderate (18%) or high (2%) quality according to GRADE. The most common domains provoking downgrading of the evidence were risk of bias (study limitations) and imprecision indicating the need for larger and higher quality randomized clinical trials to inform clinical decisions. The lack of robust evidence underpinning dental procedures should prompt a concerted drive to conduct funded, high quality clinical trials in order to improve the quality of the evidence for accepted but often unproven procedures.

## Supporting Information

S1 Fig Number of included non-Cochrane and Cochrane reviews by year of publications. (TIF)

S2 Fig Heterogeneity (I-square %) for non-Cochrane and Cochrane included meta-analyses. (TIF)

S1 File Detailed description of assessing the quality of the evidence per GRADE domain. (DOCX)

S1 Table Collected information per systematic review at review, meta-analysis, trial level and GRADE assessment. (DOCX)

S2 Table GRADE assessment at the selected meta-analysis level. Mixed indicates inclusion of both randomized and non-randomised studies in the meta-analysis.
(DOCX)

S3 Table Frequencies and percentages of included review characteristics for Cochrane and non-Cochrane reviews at review level. (DOCX)

S4 Table Frequencies and percentages of characteristics of selected meta-analyses for detailed assessment for 41 Cochrane and 50 not Cochrane reviews at meta-analysis level. (DOCX)
