# Supplementary figures and images for: The Quality of the Evidence According to GRADE Is Predominantly Low or Very Low in Oral Health Systematic Reviews
Source: PLoS One. 2015 Jul 10;10(7):e0131644. doi: 10.1371/journal.pone.0131644 (PMC4498810; doi:10.1371/journal.pone.0131644)

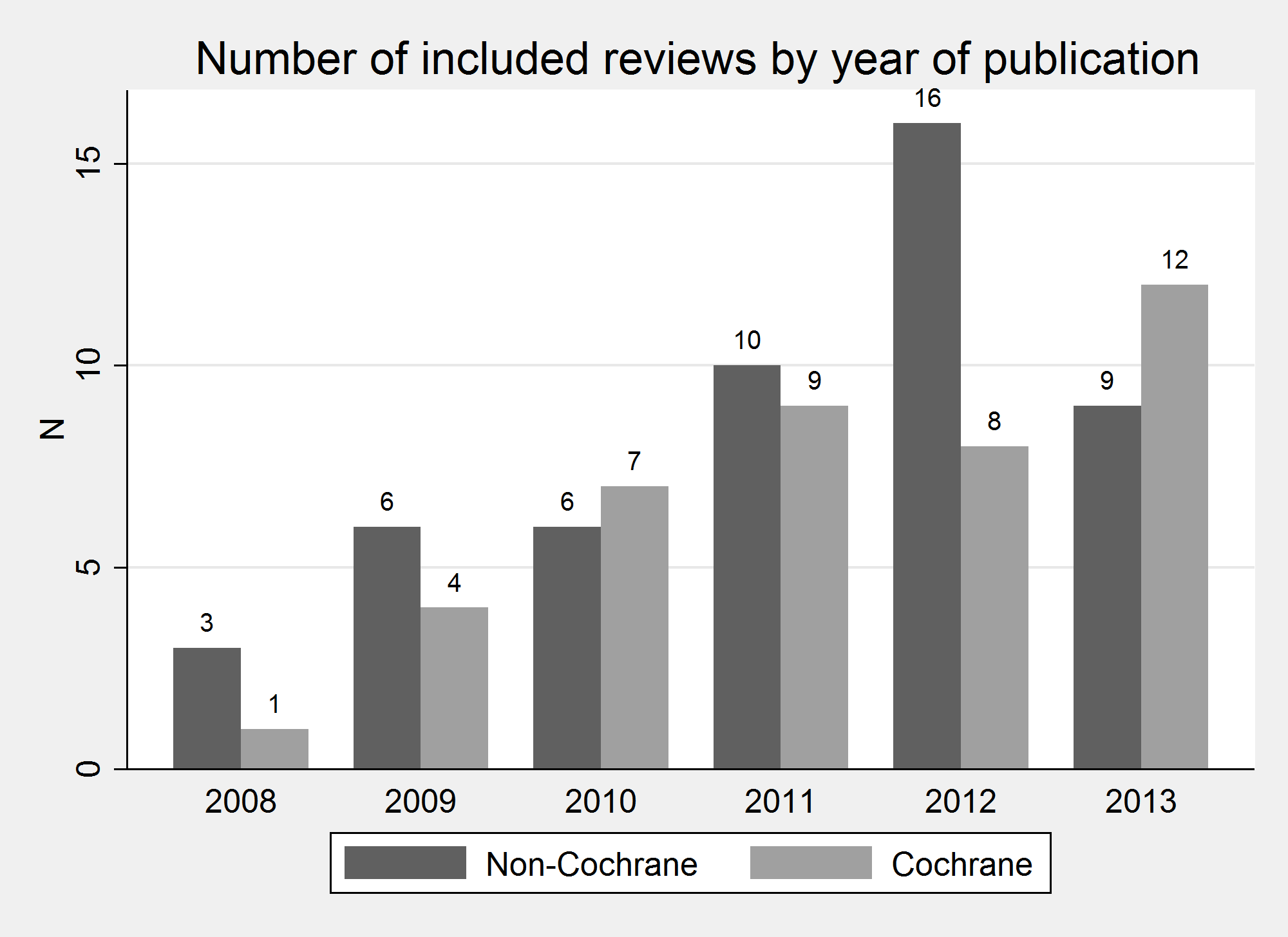

Supplement: S1 Fig — (TIF) [file pone.0131644.s001.tif]

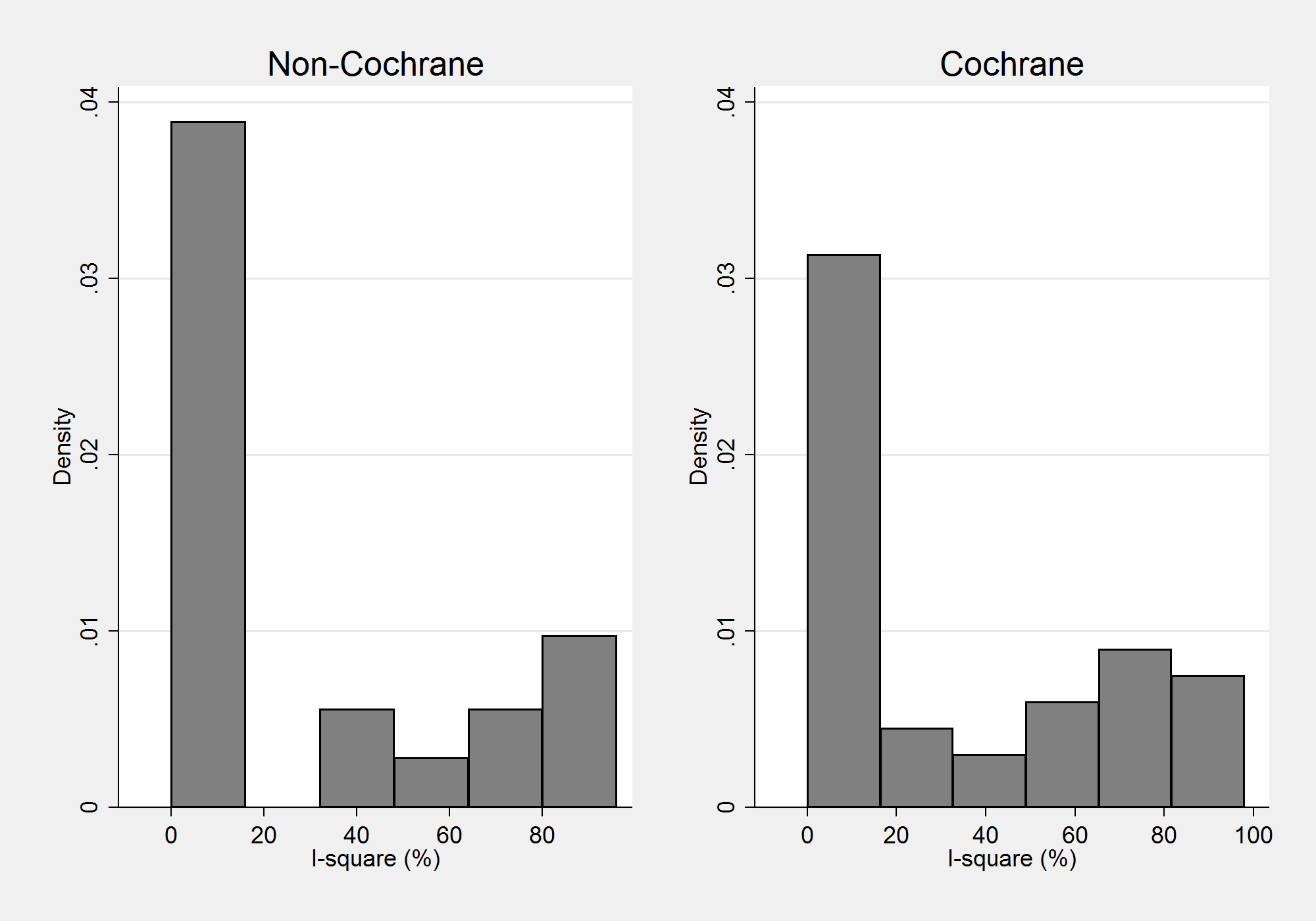

Supplement: S2 Fig — (TIF) [file pone.0131644.s002.tif]
