# Supplementary material for: The Quality of the Evidence According to GRADE Is Predominantly Low or Very Low in Oral Health Systematic Reviews
Source: PLoS One. 2015 Jul 10;10(7):e0131644. doi: 10.1371/journal.pone.0131644 (PMC4498810; doi:10.1371/journal.pone.0131644)
Supplement: S1 File — (DOCX) [file pone.0131644.s003.docx]

**Detailed explanation for assessing the level of evidence using the GRADE approach** [1–5]

**Limitations in study design (Risk of bias)** was assessed in the following domains: allocation concealment, blinding (participants and assessors), especially in the presence of subjective outcomes, attrition, selective outcome reporting and other limitations ( Stopping early for benefit, use of non-validated outcome measures such as patient reported outcomes, carry-over effect in cross-over designs and recruitment bias in cluster randomized designs). The assessments for the study limitations in the above domains was be based on the Cochrane Collaborations’ risk of bias tool recommendations)[6] . A rating of high, unclear or low risk of bias was assigned per domain, per trial overall and across all trials included in the selected met-analysis. Overall trial rating as high, unclear or low was done as follows: if one or more domains at unclear or high ROB declare as unclear or high ROB respectively, otherwise rating is low risk of bias. Across studies, a summary assessment is rated as low risk of bias when most information is from studies at low risk of bias, unclear risk of bias when most information (amount of information: sample size, number of events etc and not the number of studies) is from studies at low or unclear risk of bias, and high risk of bias when the proportion of information is from studies at high risk of bias sufficient to affect the interpretation of the results. Consequently, for low overall risk of bias no downgrade was implemented and for serious or very serious a 1 and 2 levels of downgrading was applied respectively.

**Inconsistency:** In the presence of heterogeneity or variability of results across included studies in the assessed meta-analysis, failure of plausible explanation for the observed heterogeneity is a reason for downgrading for inconsistency. Study methods and associated bias, variability of point estimates, confidence interval overlap and statistical criteria such as heterogeneity tests (p-value for heterogeneity and I^2^) were considered during the assessment. A rough guideline, however not restrictive, given the effect of sample size on the value of I^2^, would be: I^2^<40% is low, 30-60% may be moderate, 50-90% may be substantial, and 75-100% is regarded as considerable[7]. Minor difference in direction with overlapping confidence intervals will not result in a downgrade. A 3-point rating was utilized corresponding to no inconsistency, serious inconsistency, very serious inconsistency.

**Indirectness:** Reasons for a change in the rating of indirectness included no head-to-head comparisons and evidence derived from studies with different participants, interventions and outcomes to the question being addressed by the SR. A 3-point rating was be utilized corresponding to no indirectness, serious indirectness, very serious indirectness. Surrogate outcomes generally result in a 1-point downgrade, whereas additional issues in the other areas (participants, interventions, indirect comparisons) resulted in a 2- point downgrade.

**Imprecision**: Studies of small size and limited number of events result in imprecise estimates with wide confidence intervals. The optimal information size (OIS) criterion suggest that if the total number of patients included in the systematic review is less than the number of patients generated by a conventional sample calculation for a single adequately powered trial, a possible downgrade due to the likelihood of imprecision should be considered. The OIS guidelines according to GRADE for potentially downgrading the quality of the evidence are discussed below for binary and continuous outcomes:

Dichotomous outcomes

-total sample size is lower than the calculated optimal information size (OIS), unless sample size is very large (at least 2000 or perhaps 4000 patients).

-If the 95% CI excludes a relative risk of 1 and the total number of events exceeds the OIS, precision criterion is considered adequate. If the 95% confidence intervals ranges from unimportant to important benefits and from unimportant to important harms, GRADE recommends downgrading the quality of evidence when appreciable benefit or harm are of 25% relative risk reduction or relative risk increase, respectively.

-An exception exists if event rates are very low. In this case 95% CIs around relative effects can be very wide; however, 95% CIs around absolute effects may be narrow. In this scenario the quality of evidence may not be downgraded for precision. Additionally, if the number of patients is sufficiently large, it is probable that prognostic balance has been achieved and downgrading based on imprecision is not appropriate. The inference of unimportance should be considered over the appropriate follow-up duration, since a small number of events over a short follow-up may be misleading.

-If the sample size is large enough and there is an apparent treatment effect with a satisfactory CI there is no need to apply the OIS.

-SR quality of the evidence was not downgraded based on the balance between desirable and undesirable effects; this decision belongs to the guideline developers. Therefore, precision judgment should not be focused on the threshold indicating the clinical importance of the effect but on OIS. If OIS is not met, a downgrade is warranted unless the sample size is very large. If the criterion is met and the 95% CI around the effect excludes 1 there is no need to downgrade for imprecision

Continuous outcomes

-95% CI includes no effect and upper and lower CI bounds cross the minimal important clinical difference (MID) for harm or benefit

-if the MID is not known or use of different outcome measures is required for calculation of an effect size, GRADE recommends downgrading if the upper or lower confidence limit crosses an effect size of 0.5 in either direction.

-use OIS

Again a 3-point scale was utilized corresponding to no imprecision, serious imprecision, and very serious imprecision. Grading for imprecision was outlined earlier in detail.

**Publication bias**: Inclusion of small studies only in combination with industry sponsorship resulted in a downgrade. Publication bias was assessed by examining search methods and funnel plot asymmetry (if available). Industry sponsorship or suspicion of industry sponsorship and/or other conflicts of interest may also increase the likelihood of downgrade. Additional parameters that may influence the decision to downgrade include inclusion of small studies only, a relatively recent RCT or a number of RCTs assessing a new treatment and a less than comprehensive search for studies; for example, failing to search for unpublished trials. A 2-point rating was assigned representing either undetected or strongly suspected publication bias. Absence of publication bias is difficult to confirm; it is similarly difficult to decide on a threshold at which to downgrade in view of publication bias. In view of these challenges, GRADE suggests use of the terms ‘‘undetected’’ and ‘‘strongly suspected’’ to describe the risk of publication bias. Given the uncertainty in the presence or absence of publication bias, GRADE suggests rating down a maximum of one level (rather than two) for suspicion of publication bias. For Cochrane reviews there is a trial search coordinator and therefore we assumed that the search was sufficient. We examined the publication bias section and downgraded if the authors stated suspicion on publication bias. For non-Cochrane reviews we considered searched databases, gray literature, reference hand search and author’s statements in the review in order to decide on downgrading on this **domain.**

References

[1] Balshem H, Helfand M, Schünemann HJ, Oxman AD, Kunz R, Brozek J, et al. GRADE guidelines: 3. Rating the quality of evidence. J Clin Epidemiol 2011;64:401–6. doi:10.1016/j.jclinepi.2010.07.015.

[2] Guyatt GH, Oxman AD, Kunz R, Woodcock J, Brozek J, Helfand M, et al. GRADE guidelines: 7. Rating the quality of evidence--inconsistency. J Clin Epidemiol 2011;64:1294–302. doi:10.1016/j.jclinepi.2011.03.017.

[3] Guyatt GH, Oxman AD, Kunz R, Woodcock J, Brozek J, Helfand M, et al. GRADE guidelines: 8. Rating the quality of evidence--indirectness. J Clin Epidemiol 2011;64:1303–10. doi:10.1016/j.jclinepi.2011.04.014.

[4] Guyatt GH, Oxman AD, Montori V, Vist G, Kunz R, Brozek J, et al. GRADE guidelines: 5. Rating the quality of evidence--publication bias. J Clin Epidemiol 2011;64:1277–82. doi:10.1016/j.jclinepi.2011.01.011.

[5] Guyatt GH, Oxman AD, Kunz R, Brozek J, Alonso-Coello P, Rind D, et al. GRADE guidelines 6. Rating the quality of evidence--imprecision. J Clin Epidemiol 2011;64:1283–93. doi:10.1016/j.jclinepi.2011.01.012.

[6] Higgins JPT, Altman DG, Gøtzsche PC, Jüni P, Moher D, Oxman AD, et al. The Cochrane Collaboration’s tool for assessing risk of bias in randomised trials. BMJ 2011;343:d5928.

[7] Deeks JJ, Higgins JPT, Altman DG (editors). Chapter 9: Analysing data and undertaking meta-analyses. In: Higgins JPT, Green S (editors). Cochrane Handbook for Systematic Reviews of Interventions Version 5.1.0 (updated March 2011). The Cochrane Collaboration, 2011. Available from www.cochrane-handbook.org, n.d.
