# Supplementary material for: The Quality of the Evidence According to GRADE Is Predominantly Low or Very Low in Oral Health Systematic Reviews
Source: PLoS One. 2015 Jul 10;10(7):e0131644. doi: 10.1371/journal.pone.0131644 (PMC4498810; doi:10.1371/journal.pone.0131644)
Supplement: S1 Table — (DOCX) [file pone.0131644.s004.docx]

|  | **Collected Information per Systematic Review** |
| --- | --- |
| **Review level** | **Journal title** |
|  | **Name of first author** |
|  | **SR title** |
|  | **Year of SR publication** |
|  | **Specialty of journal** |
|  | **doi** |
|  | **Country of first author** |
|  | **Continent of first author**: Europe; Americas; Asia & other |
|  | **Number of authors** |
|  | **Involvement of methodologist** from affiliations, titles, methods-all Cochrane received a yes |
|  | **Involvement of one or more universities:** SR conducted within a single university/setting or with authors from multiple universities |
|  | **Inclusion of meta-analysis** in the SR or not |
|  | **Exclusion reasons** of SR from full data extraction |
|  | **Interventional or not** interventional SR |
|  | **GRADE table** provided by SR authors or not |
|  | **List of SR outcomes** includes treatment **benefits** or not |
|  | **List of SR outcomes** includes treatment **harms** or not |
|  | **Number of non-randomized trials** included in the SR |
|  | **Number of cases-series** included in the SR |
|  | **Number of observational studies** (cohort, case-control) included in the SR-cross-sectional excluded |
|  | **Number of parallel RCTs** included in the SR |
|  | **Number of cross-over RCTs** included in the SR |
|  | **Number of cluster RCTs** included in the SR |
|  | **Number of split-mouth RCTs** included in the SR |
|  | **Total number of studies** included in SR |
|  | **Total number of meta-analyses** included in SR |
| **Meta-analysis level** | **Number of trials** in selected meta-analysis |
|  | **Identification of meta-analysis** selected for full data extraction |
|  | **Applied intervention** in the selected meta-analysis |
|  | **Category of applied intervention** in the selected meta-analysis |
|  | **Control therapy** applied in the selected meta-analysis |
|  | **Category of applied control** in the selected meta-analysis |
|  | **Outcome** for selected meta-analysis |
|  | **Type of outcome**: subjective or objective |
|  | **Outcome scale**: continuous or binary |
|  | **Approach implemented** by SR authors to assess methodological quality: i.e. Cochrane ROB; Jadad scale |
|  | **Overall risk of bias assessment** at meta-analysis level: high; unclear; low: if one or more domains at high or unclear ROB declare as high or unclear ROB and low if all trials at low ROB |
|  | **Total sample size** when sample size per group break down was not available |
|  | **Intervention mean effect** per trial included in the selected meta-analysis |
|  | **Intervention standard deviation** (sd) of the mean effect per trial included in the selected meta-analysis |
|  | **Intervention sample size** per trial included in the selected meta-analysis |
|  | **Control mean effect** per trial included in the selected meta-analysis |
|  | **Control standard deviation** (sd) of the mean effect per trial included in the selected meta-analysis |
|  | **Control sample size** per trial included in the selected meta-analysis |
|  | **Intervention events** per trial included in the selected meta-analysis |
|  | **Intervention sample size** per trial included in the selected meta-analysis |
|  | **Control events** per trial included in the selected meta-analysis |
|  | **Control sample size** per trial included in the selected meta-analysis |
|  | **Model** used for meta-analysis: fixed; random |
|  | **Effect size value** per trial included in the selected meta-analysis and pooled |
|  | **Effect size type**: OR; RR ;HR; MD ; SMD |
|  | **Lower CI bound** of effect size value per trial included in the selected meta-analysis and pooled |
|  | **Upper CI bound** of effect size value per trial included in the selected meta-analysis and pooled |
|  | ***I^2^* value** for statistical heterogeneity |
|  | **Accounting for paired data** in the case of inclusion of split-mouth designs: not discussed or accounted for in the analysis; discussed and/or accounted for in the analysis |
|  | **Accounting for clustering effects** in the case of inclusion of clustered designs: not discussed or accounted for in the analysis; discussed and/or accounted for in the analysis in the trials |
|  |  |
| **Trial level** | **Study design** of trials included in the selected meta-analysis: parallel; split-mouth; cross-over; |
|  | **Study type** included in the meta-analysis: RCT; CCT ; case-series; cohort; case-control |
|  | **Author's name** of trials included in the selected meta-analysis |
|  | **Allocation concealment** risk of bias (ROB) assessment: high; unclear; low |
|  | **Blinding** risk of bias assessment: high; unclear; low |
|  | **Attrition** risk of bias assessment: high; unclear; low |
|  | **Selective outcome reporting** risk of bias assessment: high; unclear; low |
|  | **Other limitations** risk of bias assessment [stopping early for benefit; non- validated outcome measures; carry-over effect; recruitment bias in clustered trials] : high; unclear; low |
|  | **Overall risk of bias** assessment at trial level: high; unclear; low: if one or more domains at unclear or high ROB declare as unclear or high ROB and low otherwise |
| **GRADE assessment** | **Study limitations:** If most trials at low ROB=no downgrade for limitations of design; If most trials at unclear ROB= downgrade for limitations of design by 1 level; If most trials at high ROB= downgrade for limitations of design by 2 levels. |
|  | **Inconsistency:**  1 or 2 levels of downgrade depending on clinical & methodological heterogeneity (PICO), statistical heterogeneity, Confidence interval overlap |
|  | **Indirectness:**  1 or 2 levels of downgrade depending on whether or not head-to-head comparisons were used |
|  | **Imprecision:** 1 or 2 levels of downgrade depending on statistical heterogeneity, CI overlap and inclusion of benefits and harms in the CI |
|  | **Publication bias**: suspected or unsuspected based on completeness of search strategy, formal statistical assessment and SR authors’ comments |
|  | **Outcome Importance:** not important; important but not critical; critical. Refers to selected outcome and based on importance relative to intervention. i.e. implant failure critical but clinical attachment loss may be important but not |
|  | **Effect size:** if effect is strong applicable for observational studies RR>2 or <0.5; RR>5 or RR<0.2 |
|  | **GRADE rating in this review**: High, moderate, low, very low |
|  | **GRADE reported by SR authors** (if provided): High, moderate, low, very low |
| RCTs=Randomized controlled trials; CCT=Controlled clinical trial implying non-randomized; SR=Systematic review; ROB=Risk of bias; CI=Confidence interval; OR=Odds ratio; Risk ratio ;Hazard ratio; Mean difference ; Standardized mean difference | |

**S1 Table.**
