# Supplementary material for: The Quality of the Evidence According to GRADE Is Predominantly Low or Very Low in Oral Health Systematic Reviews
Source: PLoS One. 2015 Jul 10;10(7):e0131644. doi: 10.1371/journal.pone.0131644 (PMC4498810; doi:10.1371/journal.pone.0131644)
Supplement: S2 Table — Mixed indicates inclusion of both randomized and non-randomised studies in the meta-analysis. (DOCX) [file pone.0131644.s005.docx]

| **author** | **Study type** | **Study limitations** | **inconsistency** | **indirectness** | **Imprecision** | **Publication bias** | **GRADE** | **GRADE in SR** |
| --- | --- | --- | --- | --- | --- | --- | --- | --- |
| Chen[1] | Mixed | serious | no | no | serious | undetected | low | nr |
| Fleming[2] | Randomized | serious | no | no | no | undetected | high | high |
| Fleming[3] | Randomized | no | no | no | no | undetected | high | high |
| Kaklamanos[4] | Randomized | serious | no | no | serious | undetected | low | nr |
| Marsico[5] | Randomized | serious | no | no | no | undetected | moderate | nr |
| Santos[6] | Randomized | very serious | no | no | no | undetected | low | nr |
| Alsabeeha[7] | Mixed | very serious | no | no | very serious | undetected | very low | nr |
| Del Fabbro[8] | Mixed | very serious | no | no | no | undetected | moderate | nr |
| Del Fabbro [9] | Non-randomized | very serious | no | no | very serious | undetected | low | nr |
| Safii[10] | Non-randomized | very serious | no | no | serious | undetected | low | nr |
| Marathiotou[11] | Non-randomized | very serious | no | no | no | undetected | low | nr |
| Mickenautsch[12] | Non-randomized | very serious | no | no | serious | undetected | low | nr |
| Shabazfar[13] | Mixed | very serious | serious | no | serious | detected | very low | nr |
| Atieh[14] | Mixed | very serious | no | no | no | undetected | moderate | nr |
| Emami[15] | Randomized | serious | serious | no | serious | undetected | very low | nr |
| Rocuzzo[16] | Randomized | serious | no | no | serious | detected | low | nr |
| Sanz [17] | Mixed | very serious | no | no | very serious | undetected | very low | nr |
| Thoma[18] | Randomized | serious | serious | no | very serious | detected | very low | nr |
| Agarwal[19] | Randomized | serious | no | no | serious | undetected | low | nr |
| Ashley[20] | Randomized | serious | no | no | serious | undetected | low | moderate |
| Bessel[21] | Randomized | serious | no | no | serious | undetected | low | nr |
| Chambrone[22] | Randomized | very serious | serious | no | serious | undetected | very low | nr |
| Cooper[23] | Randomized | serious | no | no | serious | undetected | low | low |
| Daly[24] | Randomized | serious | no | no | no | undetected | moderate | moderate |
| Deacon[25] | Randomized | serious | no | no | no | undetected | moderate | nr |
| Eberhard[26] | Randomized | serious | no | no | no | undetected | moderate | nr |
| Esposito1[27] | Randomized | serious | no | no | very serious | undetected | low | nr |
| Esposito2[28] | Randomized | serious | serious | no | no | undetected | low | low |
| Esposito3[29] | Randomized | serious | no | no | serious | undetected | low | moderate |
| Esposito4[30] | Randomized | serious | no | no | serious | undetected | low | low |
| Esposito5[31] | Randomized | serious | no | no | serious | undetected | very low | nr |
| Esposito6[32] | Randomized | serious | no | no | very serious | undetected | very low | nr |
| Esposito7[33] | Randomized | very serious | no | no | very serious | undetected | very low | nr |
| Esposito8[34] | Randomized | serious | no | no | no | undetected | moderate | moderate |
| Furness1[35] | Randomized | serious | no | serious | no | undetected | very low | very low |
| Furness2[36] | Randomized | serious | no | no | serious | undetected | low | low |
| Furness3[37] | Randomized | very serious | no | serious | no | undetected | low | nr |
| Glenny1[38] | Randomized | serious | no | no | no | undetected | moderate | nr |
| Glenny2[39] | Randomized | very serious | serious | serious | no | undetected | low | low |
| Hu[40] | Randomized | serious | no | no | serious | undetected | low | low |
| Jambi[41] | Randomized | serious | serious | no | serious | undetected | very low | very low |
| Tubert-Jeannin[42] | Randomized | serious | no | no | no | undetected | moderate | nr |
| Lodi[43] | Randomized | very serious | no | no | no | undetected | moderate | moderate |
| Marihno[44] | Randomized | serious | no | no | no | undetected | moderate | moderate |
| Martharou[45] | Randomized | serious | serious | no | no | undetected | low | nr |
| Millett[46] | Randomized | no | serious | no | serious | undetected | low | nr |
| Nasser[47] | Randomized | serious | no | no | very serious | undetected | low | nr |
| Ricketts[48] | Randomized | serious | no | no | no | undetected | moderate | moderate |
| Rigon[49] | Randomized | very serious | no | no | serious | undetected | very low | nr |
| Ahovuo-Saloranta[50] | Randomized | no | no | no | no | undetected | moderate | moderate |
| Sambunjak[51] | Randomized | serious | serious | no | serious | undetected | very low | very low |
| Shi[52] | Randomized | serious | no | no | no | undetected | moderate | moderate |
| Simpson[53] | Randomized | serious | no | no | serious | undetected | low | low |
| Thiruvenkatachari[54] | Randomized | serious | serious | no | no | undetected | low | low |
| Thongprasom[55] | Randomized | very serious | no | no | serious | undetected | low | nr |
| Watkinson[56] | Randomized | serious | serious | no | no | undetected | low | low |
| Worthington[57] | Randomized | very serious | serious | no | no | undetected | very low | nr |
| Worthington[58] | Randomized | very serious | no | no | serious | undetected | very low | nr |
| Worthington[59] | Randomized | serious | no | no | serious | undetected | low | low |
| Atieh[60] | Randomized | serious | no | no | serious | undetected | low | nr |
| Annibali[61] | Randomized | very serious | serious | no | no | undetected | low | nr |
| Bouziane[62] | Randomized | very serious | serious | no | no | detected | very low | nr |
| Cairo[63] | Randomized | very serious | no | no | no | undetected | low | nr |
| Chambrone[64] | Randomized | serious | no | no | serious | detected | low | nr |
| Eberhard[65] | Randomized | serious | no | no | very serious | undetected | low | nr |
| Kunnen[66] | Randomized | serious | no | no | serious | undetected | low | nr |
| Sgolastra[67] | Randomized | serious | serious | no | no | undetected | low | nr |
| He[68] | Randomized | serious | no | no | serious | undetected | low | nr |
| Jung[69] | Randomized | serious | no | no | serious | undetected | low | nr |
| Katyal[70] | Randomized | serious | no | no | no | undetected | moderate | nr |
| Schwendicke[71] | Randomized | very serious | serious | no | very serious | undetected | very low | very low |
| Cruz[72] | Randomized | very serious | very serious | no | serious | undetected | low | nr |
| Long[73] | Randomized | very serious | no | no | no | undetected | low | nr |
| Schwendicke[74] | Randomized | very serious | no | no | no | undetected | moderate | moderate |
| Stoecklin-Wasmer[75] | Randomized | very serious | serious | no | no | detected | very low | nr |
| Gillen[76] | Not reported | serious | serious | no | no | undetected | moderate | nr |
| Su[77] | Randomized | serious | no | no | serious | undetected | low | nr |
| Tsesis[78] | Randomized | serious | no | no | very serious | detected | very low | nr |
| Dan[79] | Mixed | serious | no | no | no | undetected | moderate | nr |
| Katsnelson[80] | Non-randomized | very serious | no | no | serious | undetected | very low | nr |
| Carrasco-labra[81] | Randomized | serious | serious | no | serious | undetected | very low | very low |
| Li[82] | Randomized | serious | serious | no | serious | undetected | low | nr |
| Brignardello-Petersen[83] | Randomized | very serious | no | no | serious | undetected | very low | nr |
| Yu[84] | Randomized | serious | no | no | serious | undetected | low | nr |
| atieh[85] | Mixed | very serious | no | no | very serious | detected | very low | nr |
| darby[86] | Mixed | no | no | no | serious | undetected | moderate | nr |
| koop[87] | Randomized | very serious | serious | no | serious | undetected | very low | nr |
| kotsovilis[88] | Not reported | serious | serious | no | no | undetected | low | nr |
| lin[89] | Randomized | serious | no | no | no | undetected | moderate | nr |
| sgolastra[90] | Randomized | very serious | no | no | serious | undetected | very low | nr |
| sohrabi[91] | Randomized | serious | no | no | serious | undetected | low | nr |

**S2 Table**

**References**

1. Chen SS-H, Greenlee GM, Kim J-E, Smith CL, Huang GJ. Systematic review of self-ligating brackets. Am J Orthod Dentofacial Orthop. 2010;137: 726.e1–726.e18. doi:10.1016/j.ajodo.2009.11.009

2. Fleming PS, Johal A, Pandis N. Self-etch primers and conventional acid-etch technique for orthodontic bonding: A systematic review and meta-analysis. Am J Orthod Dentofacial Orthop. 2012;142: 83–94. doi:10.1016/j.ajodo.2012.02.023

3. Padhraig S. Fleming, Eliades T, Katsaros C, Pandis N. Curing lights for orthodontic bonding: A systematic review and meta-analysis. Am J Orthod Dentofacial Orthop. 2013;143: S92–S103. doi:10.1016/j.ajodo.2012.07.018

4. Kaklamanos EG, Kalfas S. Meta-analysis on the effectiveness of powered toothbrushes for orthodontic patients. Am J Orthod Dentofacial Orthop. 2008;133: 187.e1–187.e14. doi:10.1016/j.ajodo.2007.07.015

5. Marsico E, Gatto E, Burrascano M, Matarese G, Cordasco G. Effectiveness of orthodontic treatment with functional appliances on mandibular growth in the short term. Am J Orthod Dentofacial Orthop. 2011;139: 24–36. doi:10.1016/j.ajodo.2010.04.028

6. Santos APP, Oliveira BH, Nadanovsky P. Effects of Low and Standard Fluoride Toothpastes on Caries and Fluorosis: Systematic Review and Meta-Analysis. Caries Res. 2013;47: 382–390. doi:10.1159/000348492

7. Alsabeeha N, Atieh M, Payne AGT. Loading Protocols for Mandibular Implant Overdentures: A Systematic Review with Meta-Analysis: Mandibular Overdenture Review and Meta-Analysis. Clin Implant Dent Relat Res. 2009;12: e28–e38. doi:10.1111/j.1708-8208.2009.00152.x

8. Del Fabbro M, Ceresoli V, Taschieri S, Ceci C, Testori T. Immediate Loading of Postextraction Implants in the Esthetic Area: Systematic Review of the Literature: Immediate Implant Placement and Restoration. Clin Implant Dent Relat Res. 2013; n/a–n/a. doi:10.1111/cid.12074

9. Del Fabbro M, Bellini CM, Romeo D, Francetti L. Tilted Implants for the Rehabilitation of Edentulous Jaws: A Systematic Review: Literature Review of Tilted Implants. Clin Implant Dent Relat Res. 2012;14: 612–621. doi:10.1111/j.1708-8208.2010.00288.x

10. Safii SH, Palmer RM, Wilson RF. Risk of Implant Failure and Marginal Bone Loss in Subjects with a History of Periodontitis: A Systematic Review and Meta-Analysis. Clin Implant Dent Relat Res. 2009; doi:10.1111/j.1708-8208.2009.00162.x

11. Ioannidou-Marathiotou I, Zafeiriadis AA, Papadopoulos MA. Root resorption of endodontically treated teeth following orthodontic treatment: a meta-analysis. Clin Oral Investig. 2013;17: 1733–1744. doi:10.1007/s00784-012-0860-8

12. Mickenautsch S, Yengopal V, Banerjee A. Retention of orthodontic brackets bonded with resin-modified GIC versus composite resin adhesives—a quantitative systematic review of clinical trials. Clin Oral Investig. 2012;16: 1–14. doi:10.1007/s00784-011-0626-8

13. Shabazfar N, Daubländer M, Al-Nawas B, Kämmerer PW. Periodontal intraligament injection as alternative to inferior alveolar nerve block—meta-analysis of the literature from 1979 to 2012. Clin Oral Investig. 2014;18: 351–358. doi:10.1007/s00784-013-1113-1

14. Atieh MA, Payne AGT, Duncan WJ, Cullinan MP. Immediate restoration/loading of immediately placed single implants: is it an effective bimodal approach? Clin Oral Implants Res. 2009;20: 645–659. doi:10.1111/j.1600-0501.2009.01725.x

15. Emami E, Heydecke G, Rompré PH, de Grandmont P, Feine JS. Impact of implant support for mandibular dentures on satisfaction, oral and general health-related quality of life: a meta-analysis of randomized-controlled trials. Clin Oral Implants Res. 2009; doi:10.1111/j.1600-0501.2008.01693.x

16. Roccuzzo M, Bonino F, Gaudioso L, Zwahlen M, Meijer HJA. What is the optimal number of implants for removable reconstructions? A systematic review on implant-supported overdentures. Clin Oral Implants Res. 2012;23: 229–237. doi:10.1111/j.1600-0501.2012.02544.x

17. Sanz I, Garcia-Gargallo M, Herrera D, Martin C, Figuero E, Sanz M. Surgical protocols for early implant placement in post-extraction sockets: a systematic review. Clin Oral Implants Res. 2012;23: 67–79. doi:10.1111/j.1600-0501.2011.02339.x

18. Thoma DS, Benić GI, Zwahlen M, Hämmerle CHF, Jung RE. A systematic review assessing soft tissue augmentation techniques. Clin Oral Implants Res. 2009;20: 146–165. doi:10.1111/j.1600-0501.2009.01784.x

19. Aggarwal VR, Lovell K, Peters S, Javidi H, Joughin A, Goldthorpe J. Psychosocial interventions for the management of chronic orofacial pain. Cochrane Database Syst Rev. 2011; CD008456. doi:10.1002/14651858.CD008456.pub2

20. Ashley PF, Parekh S, Moles DR, Anand P, Behbehani A. Preoperative analgesics for additional pain relief in children and adolescents having dental treatment. Cochrane Database Syst Rev. 2012;9: CD008392. doi:10.1002/14651858.CD008392.pub2

21. Bessell A, Hooper L, Shaw WC, Reilly S, Reid J, Glenny A-M. Feeding interventions for growth and development in infants with cleft lip, cleft palate or cleft lip and palate. Cochrane Database Syst Rev. 2011; CD003315. doi:10.1002/14651858.CD003315.pub3

22. Chambrone L, Sukekava F, Araújo MG, Pustiglioni FE, Chambrone LA, Lima LA. Root coverage procedures for the treatment of localised recession-type defects. Cochrane Database Syst Rev. 2009; CD007161. doi:10.1002/14651858.CD007161.pub2

23. Cooper AM, O’Malley LA, Elison SN, Armstrong R, Burnside G, Adair P, et al. Primary school-based behavioural interventions for preventing caries. Cochrane Database Syst Rev. 2013;5: CD009378. doi:10.1002/14651858.CD009378.pub2

24. Daly B, Sharif MO, Newton T, Jones K, Worthington HV. Local interventions for the management of alveolar osteitis (dry socket). Cochrane Database Syst Rev. 2012;12: CD006968. doi:10.1002/14651858.CD006968.pub2

25. Deacon SA, Glenny A-M, Deery C, Robinson PG, Heanue M, Walmsley AD, et al. Different powered toothbrushes for plaque control and gingival health. Cochrane Database Syst Rev. 2010; CD004971. doi:10.1002/14651858.CD004971.pub2

26. Eberhard J, Jepsen S, Jervøe-Storm P-M, Needleman I, Worthington HV. Full-mouth disinfection for the treatment of adult chronic periodontitis. Cochrane Database Syst Rev. 2008; CD004622. doi:10.1002/14651858.CD004622.pub2

27. Esposito M, Grusovin MG, Felice P, Karatzopoulos G, Worthington HV, Coulthard P. Interventions for replacing missing teeth: horizontal and vertical bone augmentation techniques for dental implant treatment. Cochrane Database Syst Rev. 2009; CD003607. doi:10.1002/14651858.CD003607.pub4

28. Esposito M, Grusovin MG, Papanikolaou N, Coulthard P, Worthington HV. Enamel matrix derivative (Emdogain(R)) for periodontal tissue regeneration in intrabony defects. Cochrane Database Syst Rev. 2009; CD003875. doi:10.1002/14651858.CD003875.pub3

29. Esposito M, Grusovin MG, Chew YS, Coulthard P, Worthington HV. Interventions for replacing missing teeth: 1- versus 2-stage implant placement. Cochrane Database Syst Rev. 2009; CD006698. doi:10.1002/14651858.CD006698.pub2

30. Esposito M, Grusovin MG, Rees J, Karasoulos D, Felice P, Alissa R, et al. Interventions for replacing missing teeth: augmentation procedures of the maxillary sinus. Cochrane Database Syst Rev. 2010; CD008397. doi:10.1002/14651858.CD008397

31. Esposito M, Grusovin MG, Polyzos IP, Felice P, Worthington HV. Interventions for replacing missing teeth: dental implants in fresh extraction sockets (immediate, immediate-delayed and delayed implants). Cochrane Database Syst Rev. 2010; CD005968. doi:10.1002/14651858.CD005968.pub3

32. Esposito M, Maghaireh H, Grusovin MG, Ziounas I, Worthington HV. Interventions for replacing missing teeth: management of soft tissues for dental implants. Cochrane Database Syst Rev. 2012;2: CD006697. doi:10.1002/14651858.CD006697.pub2

33. Esposito M, Grusovin MG, Worthington HV. Interventions for replacing missing teeth: treatment of peri-implantitis. Cochrane Database Syst Rev. 2012;1: CD004970. doi:10.1002/14651858.CD004970.pub5

34. Esposito M, Grusovin MG, Worthington HV. Interventions for replacing missing teeth: antibiotics at dental implant placement to prevent complications. Cochrane Database Syst Rev. 2013;7: CD004152. doi:10.1002/14651858.CD004152.pub4

35. Furness S, Glenny A-M, Worthington HV, Pavitt S, Oliver R, Clarkson JE, et al. Interventions for the treatment of oral cavity and oropharyngeal cancer: chemotherapy. Cochrane Database Syst Rev. 2011; CD006386. doi:10.1002/14651858.CD006386.pub3

36. Furness S, Bryan G, McMillan R, Birchenough S, Worthington HV. Interventions for the management of dry mouth: non-pharmacological interventions. Cochrane Database Syst Rev. 2013;9: CD009603. doi:10.1002/14651858.CD009603.pub3

37. Furness S, Worthington HV, Bryan G, Birchenough S, McMillan R. Interventions for the management of dry mouth: topical therapies. Cochrane Database Syst Rev. 2011; CD008934. doi:10.1002/14651858.CD008934.pub2

38. Glenny A-M, Fernandez Mauleffinch LM, Pavitt S, Walsh T. Interventions for the prevention and treatment of herpes simplex virus in patients being treated for cancer. Cochrane Database Syst Rev. 2009; CD006706. doi:10.1002/14651858.CD006706.pub2

39. Glenny A-M, Furness S, Worthington HV, Conway DI, Oliver R, Clarkson JE, et al. Interventions for the treatment of oral cavity and oropharyngeal cancer: radiotherapy. Cochrane Database Syst Rev. 2010; CD006387. doi:10.1002/14651858.CD006387.pub2

40. Hu H, Li C, Li F, Chen J, Sun J, Zou S, et al. Enamel etching for bonding fixed orthodontic braces. Cochrane Database Syst Rev. 2013;11: CD005516. doi:10.1002/14651858.CD005516.pub2

41. Jambi S, Thiruvenkatachari B, O’Brien KD, Walsh T. Orthodontic treatment for distalising upper first molars in children and adolescents. Cochrane Database Syst Rev. 2013;10: CD008375. doi:10.1002/14651858.CD008375.pub2

42. Tubert-Jeannin S, Auclair C, Amsallem E, Tramini P, Gerbaud L, Ruffieux C, et al. Fluoride supplements (tablets, drops, lozenges or chewing gums) for preventing dental caries in children. Cochrane Database Syst Rev. 2011; CD007592. doi:10.1002/14651858.CD007592.pub2

43. Lodi G, Figini L, Sardella A, Carrassi A, Del Fabbro M, Furness S. Antibiotics to prevent complications following tooth extractions. Cochrane Database Syst Rev. 2012;11: CD003811. doi:10.1002/14651858.CD003811.pub2

44. Marinho VCC, Worthington HV, Walsh T, Clarkson JE. Fluoride varnishes for preventing dental caries in children and adolescents. Cochrane Database Syst Rev. 2013;7: CD002279. doi:10.1002/14651858.CD002279.pub2

45. Lourenço-Matharu L, Ashley PF, Furness S. Sedation of children undergoing dental treatment. Cochrane Database Syst Rev. 2012;3: CD003877. doi:10.1002/14651858.CD003877.pub4

46. Millett DT, Mandall NA, Mattick RC, Hickman J, Glenny A-M. Adhesives for bonded molar tubes during fixed brace treatment. Cochrane Database Syst Rev. 2011; CD008236. doi:10.1002/14651858.CD008236.pub2

47. Nasser M, Pandis N, Fleming PS, Fedorowicz Z, Ellis E, Ali K. Interventions for the management of mandibular fractures. Cochrane Database Syst Rev. 2013;7: CD006087. doi:10.1002/14651858.CD006087.pub3

48. Ricketts D, Lamont T, Innes NPT, Kidd E, Clarkson JE. Operative caries management in adults and children. Cochrane Database Syst Rev. 2013;3: CD003808. doi:10.1002/14651858.CD003808.pub3

49. Rigon M, Pereira LM, Bortoluzzi MC, Loguercio AD, Ramos AL, Cardoso JR. Arthroscopy for temporomandibular disorders. Cochrane Database Syst Rev. 2011; CD006385. doi:10.1002/14651858.CD006385.pub2

50. Ahovuo-Saloranta A, Forss H, Walsh T, Hiiri A, Nordblad A, Mäkelä M, et al. Sealants for preventing dental decay in the permanent teeth. Cochrane Database Syst Rev. 2013;3: CD001830. doi:10.1002/14651858.CD001830.pub4

51. Sambunjak D, Nickerson JW, Poklepovic T, Johnson TM, Imai P, Tugwell P, et al. Flossing for the management of periodontal diseases and dental caries in adults. Cochrane Database Syst Rev. 2011; CD008829. doi:10.1002/14651858.CD008829.pub2

52. Shi Z, Xie H, Wang P, Zhang Q, Wu Y, Chen E, et al. Oral hygiene care for critically ill patients to prevent ventilator-associated pneumonia. Cochrane Database Syst Rev. 2013;8: CD008367. doi:10.1002/14651858.CD008367.pub2

53. Simpson TC, Needleman I, Wild SH, Moles DR, Mills EJ. Treatment of periodontal disease for glycaemic control in people with diabetes. Cochrane Database Syst Rev. 2010; CD004714. doi:10.1002/14651858.CD004714.pub2

54. Thiruvenkatachari B, Harrison JE, Worthington HV, O’Brien KD. Orthodontic treatment for prominent upper front teeth (Class II malocclusion) in children. Status Date Ed No Change Conclus Publ In. 2013; Available: http://onlinelibrary.wiley.com/doi/10.1002/14651858.CD003452.pub3/pdf/standard

55. Thongprasom K, Carrozzo M, Furness S, Lodi G. Interventions for treating oral lichen planus. Cochrane Database Syst Rev. 2011; CD001168. doi:10.1002/14651858.CD001168.pub2

56. Watkinson S, Harrison JE, Furness S, Worthington HV. Orthodontic treatment for prominent lower front teeth (Class III malocclusion) in children. Cochrane Database Syst Rev. 2013;9: CD003451. doi:10.1002/14651858.CD003451.pub2

57. Worthington HV, Clarkson JE, Khalid T, Meyer S, McCabe M. Interventions for treating oral candidiasis for patients with cancer receiving treatment. Cochrane Database Syst Rev. 2010; CD001972. doi:10.1002/14651858.CD001972.pub4

58. Worthington HV, Clarkson JE, Bryan G, Furness S, Glenny A-M, Littlewood A, et al. Interventions for preventing oral mucositis for patients with cancer receiving treatment. Cochrane Database Syst Rev. 2011; CD000978. doi:10.1002/14651858.CD000978.pub5

59. Worthington HV, Clarkson JE, Bryan G, Beirne PV. Routine scale and polish for periodontal health in adults. Cochrane Database Syst Rev. 2013;11: CD004625. doi:10.1002/14651858.CD004625.pub4

60. Atieh MA, Atieh AH, Payne AGT, Duncan WJ. Immediate loading with single implant crowns: a systematic review and meta-analysis. Int J Prosthodont. 2009;22: 378–387.

61. Annibali S, Bignozzi I, Cristalli MP, Graziani F, La Monaca G, Polimeni A. Peri-implant marginal bone level: a systematic review and meta-analysis of studies comparing platform switching *versus* conventionally restored implants. J Clin Periodontol. 2012;39: 1097–1113. doi:10.1111/j.1600-051X.2012.01930.x

62. Bouziane A, Ahid S, Abouqal R, Ennibi O. Effect of periodontal therapy on prevention of gastric *Helicobacter pylori* recurrence: a systematic review and meta-analysis. J Clin Periodontol. 2012;39: 1166–1173. doi:10.1111/jcpe.12015

63. Cairo F, Pagliaro U, Nieri M. Treatment of gingival recession with coronally advanced flap procedures: a systematic review. J Clin Periodontol. 2008;35: 136–162. doi:10.1111/j.1600-051X.2008.01267.x

64. Chambrone L, Pannuti CM, Guglielmetti MR, Chambrone LA. Evidence grade associating periodontitis with preterm birth and/or low birth weight: II. A systematic review of randomized trials evaluating the effects of periodontal treatment: Periodontitis and adverse pregnancy outcomes. J Clin Periodontol. 2011;38: 902–914. doi:10.1111/j.1600-051X.2011.01761.x

65. Eberhard J, Jervøe-Storm P-M, Needleman I, Worthington H, Jepsen S. Full-mouth treatment concepts for chronic periodontitis: a systematic review. J Clin Periodontol. 2008;35: 591–604. doi:10.1111/j.1600-051X.2008.01239.x

66. Kunnen A, Van Doormaal JJ, Abbas F, Aarnoudse JG, Van Pampus MG, Faas MM. Review Article: Periodontal disease and pre-eclampsia: a systematic review: Periodontal disease and pre-eclampsia. J Clin Periodontol. 2010;37: 1075–1087. doi:10.1111/j.1600-051X.2010.01636.x

67. Sgolastra F, Petrucci A, Severino M, Graziani F, Gatto R, Monaco A. Adjunctive photodynamic therapy to non-surgical treatment of chronic periodontitis: a systematic review and meta-analysis. J Clin Periodontol. 2013;40: 514–526. doi:10.1111/jcpe.12094

68. He L-B, Shao M-Y, Tan K, Xu X, Li J-Y. The effects of light on bleaching and tooth sensitivity during in-office vital bleaching: A systematic review and meta-analysis. J Dent. 2012;40: 644–653. doi:10.1016/j.jdent.2012.04.010

69. Jung A, Shin B-C, Lee MS, Sim H, Ernst E. Acupuncture for treating temporomandibular joint disorders: A systematic review and meta-analysis of randomized, sham-controlled trials. J Dent. 2011;39: 341–350. doi:10.1016/j.jdent.2011.02.006

70. Katyal V. The efficacy and safety of articaine versus lignocaine in dental treatments: A meta-analysis. J Dent. 2010;38: 307–317. doi:10.1016/j.jdent.2009.12.003

71. Schwendicke F, Meyer-Lueckel H, Dörfer C, Paris S. Failure of incompletely excavated teeth—A systematic review. J Dent. 2013;41: 569–580. doi:10.1016/j.jdent.2013.05.004

72. Cunha-Cruz J, Stout JR, Heaton LJ, Wataha JC, for Northwest PRECEDENT. Dentin Hypersensitivity and Oxalates: a Systematic Review. J Dent Res. 2011;90: 304–310. doi:10.1177/0022034510389179

73. Long H, Zhou Y, Liao L, Pyakurel U, Wang Y, Lai W. Coronectomy vs. Total Removal for Third Molar Extraction: A Systematic Review. J Dent Res. 2012;91: 659–665. doi:10.1177/0022034512449346

74. Schwendicke F, Dorfer CE, Paris S. Incomplete Caries Removal: A Systematic Review and Meta-analysis. J Dent Res. 2013;92: 306–314. doi:10.1177/0022034513477425

75. Stoecklin-Wasmer C, Rutjes AWS, da Costa BR, Salvi GE, Juni P, Sculean A. Absorbable Collagen Membranes for Periodontal Regeneration: A Systematic Review. J Dent Res. 2013;92: 773–781. doi:10.1177/0022034513496428

76. Gillen BM, Looney SW, Gu L-S, Loushine BA, Weller RN, Loushine RJ, et al. Impact of the Quality of Coronal Restoration versus the Quality of Root Canal Fillings on Success of Root Canal Treatment: A Systematic Review and Meta-analysis. J Endod. 2011;37: 895–902. doi:10.1016/j.joen.2011.04.002

77. Su Y, Wang C, Ye L. Healing Rate and Post-obturation Pain of Single- versus Multiple-visit Endodontic Treatment for Infected Root Canals: A Systematic Review. J Endod. 2011;37: 125–132. doi:10.1016/j.joen.2010.09.005

78. Tsesis I, Rosen E, Tamse A, Taschieri S, Del Fabbro M. Effect of Guided Tissue Regeneration on the Outcome of Surgical Endodontic Treatment: A Systematic Review and Meta-analysis. J Endod. 2011;37: 1039–1045. doi:10.1016/j.joen.2011.05.016

79. Dan AEB, Thygesen TH, Pinholt EM. Corticosteroid Administration in Oral and Orthognathic Surgery: A Systematic Review of the Literature and Meta-Analysis. J Oral Maxillofac Surg. 2010;68: 2207–2220. doi:10.1016/j.joms.2010.04.019

80. Katsnelson A, Markiewicz MR, Keith DA, Dodson TB. Operative Management of Temporomandibular Joint Ankylosis: A Systematic Review and Meta-Analysis. J Oral Maxillofac Surg. 2012;70: 531–536. doi:10.1016/j.joms.2011.10.003

81. Carrasco-Labra A, Brignardello-Petersen R, Yanine N, Araya I, Guyatt G. Secondary Versus Primary Closure Techniques for the Prevention of Postoperative Complications Following Removal of Impacted Mandibular Third Molars: A Systematic Review and Meta-Analysis of Randomized Controlled Trials. J Oral Maxillofac Surg. 2012;70: e441–e457. doi:10.1016/j.joms.2012.03.017

82. Li C, Zhang Y, Lv J, Shi Z. Inferior or Double Joint Spaces Injection Versus Superior Joint Space Injection for Temporomandibular Disorders: A Systematic Review and Meta-Analysis. J Oral Maxillofac Surg. 2012;70: 37–44. doi:10.1016/j.joms.2011.04.009

83. Brignardello-Petersen R, Carrasco-Labra A, Araya I, Yanine N, Beyene J, Shah PS. Is Adjuvant Laser Therapy Effective for Preventing Pain, Swelling, and Trismus After Surgical Removal of Impacted Mandibular Third Molars? A Systematic Review and Meta-Analysis. J Oral Maxillofac Surg. 2012;70: 1789–1801. doi:10.1016/j.joms.2012.01.008

84. Yu SH, Beirne OR. Laryngeal Mask Airways Have a Lower Risk of Airway Complications Compared With Endotracheal Intubation: A Systematic Review. J Oral Maxillofac Surg. 2010;68: 2359–2376. doi:10.1016/j.joms.2010.04.017

85. Atieh MA, Alsabeeha NHM, Faggion CM, Duncan WJ. The Frequency of Peri-Implant Diseases: A Systematic Review and Meta-Analysis. J Periodontol. 2012; 1–15. doi:10.1902/jop.2012.120592

86. Darby IB, Morris KH. A Systematic Review of the Use of Growth Factors in Human Periodontal Regeneration. J Periodontol. 2013;84: 465–476. doi:10.1902/jop.2012.120145

87. Koop R, Merheb J, Quirynen M. Periodontal Regeneration With Enamel Matrix Derivative in Reconstructive Periodontal Therapy: A Systematic Review. J Periodontol. 2012;83: 707–720. doi:10.1902/jop.2011.110266

88. Kotsovilis S, Fourmousis I, Karoussis IK, Bamia C. A Systematic Review and Meta-Analysis on the Effect of Implant Length on the Survival of Rough-Surface Dental Implants. J Periodontol. 2009;80: 1700–1718. doi:10.1902/jop.2009.090107

89. Lin G-H, Chan H-L, Bashutski JD, Oh T-J, Wang H-L. The Effect of Flapless Surgery on Implant Survival and Marginal Bone Level: A Systematic Review and Meta-Analysis. J Periodontol. 2014;85: e91–e103. doi:10.1902/jop.2013.130481

90. Sgolastra F, Petrucci A, Gatto R, Giannoni M, Monaco A. Long-Term Efficacy of Subantimicrobial-Dose Doxycycline as an Adjunctive Treatment to Scaling and Root Planing: A Systematic Review and Meta-Analysis. J Periodontol. 2011;82: 1570–1581. doi:10.1902/jop.2011.110026

91. Sohrabi K, Saraiya V, Laage TA, Harris M, Blieden M, Karimbux N. An Evaluation of Bioactive Glass in the Treatment of Periodontal Defects: A Meta-Analysis of Randomized Controlled Clinical Trials. J Periodontol. 2012;83: 453–464. doi:10.1902/jop.2011.110347

92. Wood L, Egger M, Gluud LL, Schulz KF, Juni P, Altman DG, et al. Empirical evidence of bias in treatment effect estimates in controlled trials with different interventions and outcomes: meta-epidemiological study. BMJ. 2008;336: 601–605. doi:10.1136/bmj.39465.451748.AD
