# Supplementary material for: The Quality of the Evidence According to GRADE Is Predominantly Low or Very Low in Oral Health Systematic Reviews
Source: PLoS One. 2015 Jul 10;10(7):e0131644. doi: 10.1371/journal.pone.0131644 (PMC4498810; doi:10.1371/journal.pone.0131644)
Supplement: S3 Table — (DOCX) [file pone.0131644.s006.docx]

| **Characteristic** | **Non- Cochrane Review** | **Cochrane Reviews** | **Total** | **Odds Ratio** | **95% CIs**** | **p-value#** |
| --- | --- | --- | --- | --- | --- | --- |
|  | **N(%)** | **N(%)** | **N(%)** |  |  |  |
| **Continent of first author affiliation** |  |  |  |  |  |  |
| Americas | 10 (20%) | 1 (2%) | 11 (12%) | Reference | - |  |
| Europe | 26 (52%) | 38 (93%) | 64(70%) | 14.62 | 1.76, 121.19 | 0.01 |
| Other | 14 (28%) | 2 (5%) | 16(18%) | 1.43 | 0.11, 18.00 | 0.78 |
| **Methodologist involvement** |  |  |  |  |  |  |
| No | 33 (66%) | 0 (0%) | 33(36%) | Reference | - |  |
| Yes | 17 (34%) | 41 (100%) | 58(64%) | 73.76 | 9.79, 555.93 | <0.001 |
| **Collaboration between centers** |  |  |  |  |  |  |
| No | 26 (52%) | 7 (17%) | 33(33%) | Reference | - |  |
| Yes | 24 (48%) | 34(83%) | 58(64%) | 5.26 | 1.97, 14.09 | 0.001 |
| **GRADE assessment by SR original authors** |  |  |  |  |  |  |
| No | 45 (90%) | 17 (41%) | 62(68%) | Reference | - |  |
| Yes | 5 (10%) | 24(59%) | 29(32%) | 12.71 | 4.17, 38.69 | <0.001 |
| **At least one harm (outcome) examined** |  |  |  |  |  |  |
| No | 29 (58%) | 14(34%) | 43(47%) | Reference | - | 0.03 |
| Yes | 21 (42%) | 27(66%) | 48(53%) | 2.66 | 1.13, 6.27 |  |
| **Total** | 50(55%) | 41(45%) | 91(100%) |  |  |  |

*^#^ Pearson X^2^ test or Fisher’s exact test, * 95% Confidence intervals for unadjusted Odds Ratios*

**S3 Table**
