# Supplementary material for: The Quality of the Evidence According to GRADE Is Predominantly Low or Very Low in Oral Health Systematic Reviews
Source: PLoS One. 2015 Jul 10;10(7):e0131644. doi: 10.1371/journal.pone.0131644 (PMC4498810; doi:10.1371/journal.pone.0131644)
Supplement: S4 Table — (DOCX) [file pone.0131644.s007.docx]

| **Characteristic** | **Non- Cochrane Review** | **Cochrane Reviews** | **Total** | **Odds Ratio** | **95% CIs^##^** | **p-value** |
| --- | --- | --- | --- | --- | --- | --- |
|  | **N (%)** | **N (%)** | **N (%)** |  |  |  |
| **Study type** |  |  |  |  |  |  |
| Randomized | 34(68%) | 41 (100%) | 75 (82%) | Not estimable |  | <0.001^#^ |
| Non-randomized | 5 (10%) | 0(0%) | 5 (6%) | Not estimable |  |  |
| Mixed | 9 (18%) | 0 (0%) | 9 (10%) | Not estimable |  |  |
| Not-reported | 2 (4%) | 0 (0%) | 2 (2%) | Not estimable |  |  |
|  |  |  |  |  |  |  |
| **Model** |  |  |  |  |  |  |
| Random | 33 (66%) | 25 (61%) | 58 (66%) | Reference | - | - |
| Fixed | 17 (34%) | 16 (39%) | 33 (34%) | 1.24 | 0.53, 2.93 | 0.62 |
|  |  |  |  |  |  |  |
|  |  |  |  |  |  |  |
| **Outcome Type*** |  |  |  |  |  |  |
| Subjective | 6 (12%) | 10 (24%) | 16 (18%) | Reference | - | - |
| Objective | 44 (88%) | 31 (76%) | 75 (82%) | 2.37 | 0.78, 7.20 | 0.13 |
|  |  |  |  |  |  |  |
| **Outcome Scale** |  |  |  |  |  |  |
| Binary | 28 (56%) | 22 (54%) | 50 (56%) | Reference | - |  |
| Continuous | 19 (38%) | 15 (37%) | 34 (37%) | 1 | 0.42, 2.42 | 0.99 |
| Ordinal | 3 (6%) | 4 (10%) | 7 (7%) | 1.70 | 0.34, 8.39 | 0.52 |
| **Effect measure** |  |  |  |  |  |  |
| Hazard ratio (HR) | 0 (0%) | 2 (5%) | 2 (2%) | Not estimable |  | 0.83^#^ |
| Mean difference (MD) | 18 (36%) | 10 (24%) | 28 (31%) | Not estimable |  |  |
| Odds ratio (OR) | 9 (18%) | 4 (10%) | 13 (14%) | Not estimable |  |  |
| Preventive fraction(PF) | 0 (0%) | 2 (5%) | 2 (2%) | Not estimable |  |  |
| Risk difference (RD) | 1 (2%) | 0 (0%) | 1 (1%) | Not estimable |  |  |
| Risk ratio (RR) | 19 (38%) | 15 (37%) | 34 (37%) | Not estimable |  |  |
| Standardized mean difference (SMD) | 3 (6%) | 8 (19%) | 11 (12%) | Not estimable |  |  |
| **Total** | 50(100%) | 41(100%) | 91(100%) |  |  |  |
| **Clustering accounted/discussed**** |  |  |  |  |  |  |
| No | 25 (76%) | 2 (17%) | 27 (60%) | Reference | - | - |
| Yes | 8 (24%) | 10 (83%) | 18 (40%) | 15.62 | 2.81, 86.76 | 0.002 |
| **Total** | 33 (100%) | 12 (100%) | 45 (100%) |  |  |  |
| **Paired design accounted/discussed**** |  |  |  |  |  |  |
| No | 9 (64%) | 0 (0%) | 8 (35%) | Reference |  |  |
| Yes | 5 (36%) | 10 (100%) | 15 (65%) | 11.25 | 11.05, 541.20 | 0.02 |
| **Total** | 14 (100%) | 10 (100%) | 23 (100%) |  |  |  |

*^#^ Pearson X^2^ test or Fisher’s exact test, ^##^ 95% Confidence intervals*

* Outcome classification based on Wood et al [92] and appropriately adjusted for the selected outcomes

**Restricted to applicable meta-analyses

**S4 Table**
